# Microbiome: Living Well and Thriving With Your Bacteria

**DOI:** 10.1093/geroni/igaa057.2636

**Published:** 2020-12-16

**Authors:** Meng Wang, William Ludington

## Abstract

This symposium aims to invite speakers who are the leading experts in the field of microbiome and longevity, and select short talks from abstracts submitted by young investigators, postdoctoral fellows and graduate students. We will discuss new progress in this exciting research area and raise new questions for future studies. Confirmed speakers include Evgeny Nudler from NYU, Will Ludington from Carnegie, Paul O’Toole from University College Cork, Ireland, and Meng Wang from Baylor College of Medicine.

